# Aberrance of Zinc Metalloenzymes-Induced Human Diseases and Its Potential Mechanisms

**DOI:** 10.3390/nu13124456

**Published:** 2021-12-13

**Authors:** Yunqi Cheng, Hongping Chen

**Affiliations:** 1 Queen Mary School, Medical College, Nanchang University, Nanchang 330006, China; cyunqi@126.com; 2 Department of Histology and Embryology, Medical College, Nanchang University, Nanchang 330006, China

**Keywords:** zinc, zinc metalloenzyme, enzyme activity, human disease

## Abstract

Zinc, an essential micronutrient in the human body, is a component in over 300 enzymes and participates in regulating enzymatic activity. Zinc metalloenzymes play a crucial role in physiological processes including antioxidant, anti-inflammatory, and immune responses, as well as apoptosis. Aberrant enzyme activity can lead to various human diseases. In this review, we summarize zinc homeostasis, the roles of zinc in zinc metalloenzymes, the physiological processes of zinc metalloenzymes, and aberrant zinc metalloenzymes in human diseases. In addition, potential mechanisms of action are also discussed. This comprehensive understanding of the mechanisms of action of the regulatory functions of zinc in enzyme activity could inform novel zinc-micronutrient-supply strategies for the treatment of diseases.

## 1. Introduction

Zinc (Zn^2+^) is one of the essential trace elements in the human body and plays an extremely important role in physiological processes and pathological states. In the early 20th century, the importance of Zn^2+^ in human nutrition was controversial. Although some scholars believed that Zn^2+^ was an indispensable nutritional element for higher animals, there was no experimental proof at that time. In 1934, Todd et al. found that Zn^2+^ was essential for the development and health of rats [1]. In 1963, Prasad et al. were the first to demonstrate Zn^2+^ deficiency in the human body and found a relationship between dwarfism and Zn^2+^ deficiency, which initiated the human study of Zn^2+^ [2]. Since then, scientists have focused on the role of Zn^2+^ in human physiology, and various studies on Zn^2+^ have emerged.

As the second most abundant trace element in the human body, Zn^2+^ is responsible for the structure and catalytic activity of more than 300 enzymes [3]. The content of Zn^2+^ in the human body is generally 2–3 grams, less than 50 mg/kg [4]; ninety percent of Zn^2+^ is found in human muscles and bones [5]. Zn^2+^ performs essential functions in the human body, mainly by affecting the compositions of enzymes and proteins. First, Zn^2+^ participates in antioxidant processes to inhibit oxidative stress. Superoxide dismutase (SOD) is the main antioxidant protecting against reactive oxygen species. Zn^2+^ acts as a cofactor of SOD1, which removes free radicals in both the cytoplasm and extracellular matrix [6]. Zn^2+^ can reversibly inhibit membrane phosphodiesterase (PDE) and reduce PDE mRNA expression, which decreases the production of the inflammatory cytokines tumor necrosis factor (TNF)-alpha and interleukin (IL)-1 beta, resulting in anti-inflammatory function [7]. Zn^2+^ is also involved in regulating the immune system. Zn^2+^ helps to maintain the numbers of lymphocytes, regulatory T cells (Treg), T helper cells (Th), and cytotoxic T cells which function in defense against infection [8]. In addition, Zn^2+^ participates in cell apoptosis, and an imbalance in Zn^2+^ homeostasis can cause serious harm to health [9]. Thus, the maintenance of Zn^2+^ concentrations at normal levels is essential for human health.

Zn^2+^ metalloenzymes are a series of enzymes regulated by the structure- and activity-maintenance functions of Zn^2+^ [10]. Since these enzymes are involved in important physiological processes in humans, the disturbance of Zn^2+^ homeostasis can lead to various serious disorders, such as cardiovascular, immune system, and respiratory diseases. Zn^2+^ deficiency is a more common cause of health problems than Zn^2+^ excess. Nearly two billion people are affected by Zn^2+^ deficiency worldwide, which is more common in developing countries in Africa and Asia [11]. In developed countries, the elderly and people with chronic diseases are more likely to develop Zn^2+^ deficiency [12].

Considering the importance of Zn^2+^ in regulating Zn^2+^ metalloenzyme activity and human health, it is critical to establish the relationships among Zn^2+^, Zn^2+^ metalloenzymes and various diseases. In this review, we focus on how Zn^2+^ participates in regulating Zn^2+^ metalloenzyme activity and its influence on the physiological processes involved, as well as the enzyme dysregulation caused by Zn^2+^-homeostasis disorders in different diseases. We summarize the current evidence in the literature and extensively discuss the effect of Zn^2+^ dysregulation on Zn^2+^ metalloenzymes and common diseases, which may be beneficial for understanding the severity of Zn^2+^ homeostasis dysregulation and developing treatment strategies for diseases induced by Zn^2+^ deficiency.

## 2. Zn^2+^ Homeostasis

Since Zn^2+^ cannot be stored in large amounts in the body, a certain amount of Zn^2+^ must be consumed daily to maintain Zn^2+^ homeostasis. The estimated average requirement (EAR) of adults for Zn^2+^, recommended by the United States and Canada, ranges from 6.8 to 11 mg/day [13]. The main sources of Zn^2+^ include lean meat, liver, crustaceans, nuts, and eggs [14]. Because phytic acid acts as a chelating agent for Zn^2+^, vegetarians lack animal tissue-derived Zn^2+^ and have higher intakes of Zn^2+^-absorption inhibitors, resulting in a lower bioavailability of Zn^2+^ than that from normal diets; therefore, they need to consume more Zn^2+^ to maintain normal Zn^2+^ concentrations in the body [15]. 

The entry of dietary Zn^2+^ from the external environment into the body is mainly controlled by absorption via the gastrointestinal (GI) system. Zn^2+^ is mainly absorbed on the apical sides of enterocytes and is transported into the cytoplasm via Zn^2+^ transporters [16]. Ten members of the family of Zn^2+^ transporters and 14 members of the ZIP (Zrt- and Irt-like protein) family have been identified [17]. ZIP proteins are mainly responsible for transporting Zn^2+^ from the extracellular space or organelle into the cytoplasm. The ZIP4 protein is mainly located in the apical membrane of the intestinal epithelial cells and related tissues responsible for nutrient absorption and is responsible for transferring extracellular Zn^2+^ into cells [18]. The amount of ZIP protein is regulated by the Zn^2+^ concentration. When the Zn^2+^ concentration is increased, ZIP is removed from the cell surface through endocytosis, protecting the cells from Zn^2+^ poisoning caused by excessive Zn^2+^. Most ZIP proteins are located in cell membranes, while most ZNT (Zn^2+^ transporters) are located in the basolateral membranes of intestinal epithelial cells and are responsible for the transfer of cytoplasmic Zn^2+^ to the extracellular space; they therefore promote Zn^2+^ absorption and increase the concentration of Zn^2+^ in portal venous blood [19]. The expression of ZNT1 increases with increased dietary Zn^2+^, but Zn^2+^ deficiency does not have an effect. ZNT2 is involved in the transport of Zn^2+^ to lysosomes, and heterozygous mutations in the ZNT2 gene lead to temporary Zn^2+^ deficiency in newborns [20,21,22].

In the basolateral membranes of intestinal cells, Zn^2+^ enters the circulation mainly through ZNT1 and is then transported to the liver along the portal system. There is currently a hypothesized pathway for the ZNT4 involving vesicle-mediated exocytosis of Zn^2+^ from intestinal cells [23,24]. In addition to Zn^2+^ entering the circulatory system in an ionic state, Zn^2+^ in intestinal cells can bind to metallothionein (MT). Metallothionein, a small and cysteine-rich protein, can interact with oxides to limit Zn^2+^ absorption in enterocytes [25]. In response to Zn^2+^, the transcription of MT is rapidly promoted. It has been reported that a Zn^2+^-induced increase in MT may be involved in protection against intestinal damage caused by anti-inflammatory medicines [25,26].

After entering the circulatory system, Zn^2+^ mainly binds to albumin and is distributed to Zn^2+^-rich organs including the liver, muscles, pancreas, and prostate [27]. Zn^2+^ metabolism in eukaryotic cells is complicated. The metal-responsive element binding transcription factor 1 (MTF-1) acts as a cellular Zn^2+^ sensor that controls the expression of genes related to Zn^2+^ homeostasis, including the MT gene and genes involved in intracellular Zn^2+^ isolation and transportation. A recent study has revealed that MTF-1 reversibly binds Zn^2+^ through its Zn^2+^-finger structure and binds the metal-responsive elements in the promoters of these genes, resulting in increased transcription [28].

**Figure 1:**
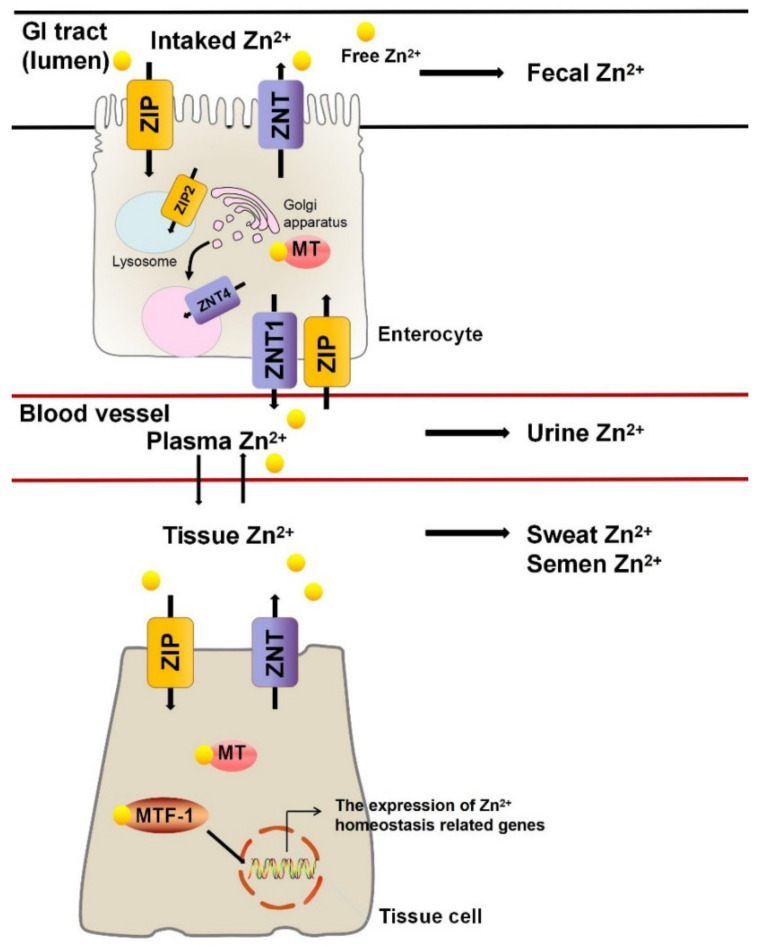
Regulation of Zn^2+^ homeostasis. Zn^2^^+^ from drinking water and diet is partially absorbed by intestinal cells in the gastrointestinal tract. Zn^2+^ enters enterocytes via ZIPs and then leaves via ZNTs into the blood circulatory system. Along with the blood, Zn^2+^ is distributed throughout the tissues. In tissue cells, MTF-1 transcription factor and MT are responsible for regulating Zn^2+^ concentration. Unused Zn^2+^ is mainly excreted in the form of feces, urine, sweat, and semen. GI, gastrointestinal; ZIP, Zrt- and Irt-like protein; ZNT, Zn^2+^ transporters; MT, metallothionein; MTF-1, metal-responsive-element-binding transcription factor-1.

Zn^2+^ is excreted from the body in the feces through the GI tract. When dietary Zn^2+^ intake is reduced, the levels of transporters responsible for Zn^2+^ absorption in the gut are increased and the expression of transporters responsible for Zn^2+^ excretion are decreased [29]. It has been reported that the pancreas plays an important role in Zn^2+^ excretion. Pancreatic acinar cells are rich in Zn^2+^-requiring proenzyme granules. ZNT2, a Zn^2+^ transporter, is distributed in proenzyme particles and participates in the transport of proenzyme granules in pancreatic acinar cells through the MTF-1 regulatory pathway, leading to the control of the excretion of Zn^2+^ from the pancreas [30]. Other physiological pathways for the loss of Zn^2+^ include sweat, urine, and semen [31]. The above findings indicate that ZNT, ZIP, MT, MTF-1, and albumin are involved in regulating the balance of Zn^2+^ concentrations, the dysfunction of which can lead to Zn^2+^ homeostasis disorders (Figure 1).

## 3. Role of Zn^2+^ and Zn^2+^ Metalloenzymes in Physiological Processes

Zn^2+^ is required by more than 300 metalloenzymes for catalytic, structural, and regulatory functions. These metalloenzymes play important roles in the human body, maintaining cell growth and normal function. In order to explore the effects of interactions between Zn^2+^ metalloenzymes and Zn^2+^ on human physiological activities, we focus on the functions of Zn^2+^ and common Zn^2+^ metalloenzymes regarding the aspects of antioxidant activity, anti-inflammatory effects, immune responses, and apoptosis.

### 3.1. Zn^2+^ Metalloenzymes Regulate Antioxidant Activity

Oxidative stress is a state of imbalance between oxidants and antioxidants, which intensifies the rate of oxidative reactions, resulting in the malfunction of redox signaling and molecular damage [32]. Although, as a divalent cation, Zn^2+^ has no physiological redox activity, it is involved in regulating antioxidant and antioxidant activity as a modulator [32]. The favorable role of Zn^2+^ in antioxidative reactions has been widely recognized. Previous studies have shown that Zn^2+^ deficiency is closely associated with increased oxidative damage to lipids, proteins, and DNA [33].

Copper/zinc superoxide dismutase (Cu/Zn-SOD) is one of the Zn^2+^ metalloenzymes that is an enzymatic antioxidant [34]. It catalyzes the conversion of oxygen free radicals into oxygen and hydrogen peroxide, which is then converted by catalase to water and oxygen. There are mainly two subtypes of Cu/Zn-SOD: one is Cu/Zn-SOD1 (SOD1), in the form of a dimer in the cell, and the other is Cu/Zn-SOD (SOD3), in the form of a tetramer in the extracellular space [35]. By binding to three histidine residues and aspartic acid in Cu/Zn-SOD, Zn^2+^ connects the structural components and provides the structural framework required for the catalytic function of SOD [36,37,38]. It has been proven that, under Zn^2+^ deficiency, the activity of SOD1 decreases, leading to an increase in reactive oxygen species (ROS) due to the loss of the cofactor [34]. Apart from acting as a cofactor, Zn^2+^ can also mask the binding site of derlin-1 on SOD1, preventing endoplasmic-reticulum stress (ERS). Derlin-1 is a member of the endoplasmic-reticulum-associated degradation (ERAD) process, and there is a Zn^2+^-masked non-exposed derlin-1 binding site in SOD1. In the Zn^2+^-restricted condition, derlin-1 binds to the binding site exposed on the Zn^2+^-free SOD1, inducing ERS [39]. Since the ERS-induced unfolded-protein response leads to an increase in ROS, it can result in excessive oxidation and oxidative stress. In addition, prolonged Zn^2+^ supplementation can enhance the activity of SOD3, preventing ROS damage [40]. Therefore, Zn^2+^ may play an antioxidant role by participating in the formation of the catalytic structure and function of SOD. 

### 3.2. Zn^2+^ Metalloenzymes Regulate Inflammation

Zn^2+^ is well known to participate in anti-inflammatory processes. Inflammation is a defensive response to tissue damage and infection. However, persistent inflammation is a cause of disease. Oxidative stress can trigger an inflammatory response, which then promotes oxidative stress by producing more ROS [41]. Neutrophils are affected by chemokines, move to the inflammatory site, and produce ROS and chemokines, promoting the infiltration of more inflammatory cells. It has been reported that, under Zn^2+^ deficiency, neutrophils have an increased ability to produce superoxide and a decreased ability to phagocytose at inflammatory sites [42]. Cu/Zn-SOD not only affects oxidative stress but is also crucial in fighting inflammation. When ROS produced by inflammatory cells accumulate, SOD is responsible for eliminating ROS that cause tissue damage. SOD is also involved in regulating neutrophil apoptosis. A previous study showed that an exogenous increase in SOD can promote the apoptosis of neutrophils, preventing the damage caused by chronic inflammation [43]. A clinical study has revealed that bovine SOD has a good effect on the treatment of skin diseases caused by persistent inflammation [44]. As Zn^2+^ can act as a cofactor to control SOD activity and then regulate the apoptosis of inflammatory cells, it plays an important role in resisting inflammation. 

Zn^2+^ can also regulate inflammation by affecting the activity of matrix metalloproteinases (MMPs). MMPs all have similar structures, and their catalytic domain contains the Zn^2+^-binding site, which is responsible for maintaining enzymatic activity. In inactive proMMPs, Zn^2+^ binds to three histidines of the catalytic metalloproteinase domain and one cysteine of the propeptide. When the Zn^2+^–cysteine bond is disrupted by other proteolytic enzymes, proMMPs becomes active with a combination of water molecules and Zn^2+^ [45,46]. A recent study has shown that MMPs can establish a chemokine gradient through the degradation and remodeling of the extracellular matrix (ECM), facilitating the movement of inflammatory cells to a damaged site [47]. The inflammatory response caused by MMP disorders can be regulated by the Zn^2+^ concentration. Zhang et al. demonstrated that the MMP concentrations in Zn^2+^-deficient mice were decreased, causing the aggregation of ECM, which aggravated the fibrosis of the spleen [48]. On the contrary, it has been reported that a Zn^2+^-supplemented diet can inhibit MMP2 and MMP9 in a lipid-disorder rabbit model, reduce the inflammatory response, and protect the liver [49]. In patients with atherosclerosis, it has been reported that the serum Zn^2+^ concentration decreased with an increase in serum MMP9 concentration [50]. A previous study has revealed that Zn^2+^ supplementation can increase the expression and activity of MMP2 and MMP8, while, under excessive Zn^2+^ supplementation, the activity of MMP significantly decreases [51]. The effect of Zn^2+^ on MMP activity may be dose dependent.

The nuclear factor kappa-light-chain enhancer of activated B cells (NF-κB) signaling pathway, a major inflammatory signaling pathway, has been shown to be negatively regulated by Zn^2+^ [41]. Phosphodiesterase (PDE), a critical component upstream of the NF-κB pathway, is responsible for controlling the concentration of cyclic adenosine monophosphate (cAMP) or cyclic guanosine monophosphate (cGMP) in cells, resulting in the effects of these second messengers being reduced. Zn^2+^ reversibly inhibits the activity of PDE by binding to two histidine and two aspartic acid residues, which leads to an increase in cGMP [52]. cGMP can cross-activate protein kinase A (PKA), resulting in the phosphorylation of Raf-1, decreasing its activity. Through this mechanism, the downstream activation of NF-κB is inhibited and the expression of its target, TNF-α, is also reduced. In addition, PDE4 is the enzyme responsible for cAMP hydrolysis, mainly in inflammatory and immune cells. Zn^2+^ regulates the inflammatory response by reversibly inhibiting the activity of PDE4 [7,53]. cAMP accumulation induced by PDE4 inhibition activates PKA, further triggering a downstream anti-inflammatory cascade, resulting in a reduced release of inflammatory factors and reduced MMP expression [54]. Thus, Zn^2+^ may be involved in the induction of anti-inflammatory responses via inhibiting PDE activity. 

### 3.3. Zn^2+^ Metalloenzymes Regulate the Immune Response

Since Zn^2+^ deficiency has been found to be significantly associated with immune-system diseases, an increasing number of studies have focused on the importance of Zn^2+^ in normal immune function [8]. In the face of the invasion of and infection by foreign organisms, the immune system exerts its defense mechanism through the interaction of immune organs, cells, and cytokines.

Various kinases, phosphatases, signaling molecules, and transcription factors constitute the major signaling pathways in the immune system. Protein kinase C (PKC) is a serine/threonine kinase, the regulatory domain of which contains a Zn^2+^-binding site. Zn^2+^ is involved in regulating PKC’s structure and facilitating the enzyme’s activity by linking to cysteine residues [55]. The PKC family plays a key role in T-cell-related immune responses. PKC θ has been reported to be involved in T-cell-receptor/CD3 activation and promotes the presentation of antigens from antigen-presenting cells (APCs) to T cells by enhancing adhesion contact between cells [56]. Under a high concentration of Zn^2+^-chelating agent, PKC activity in immune cells was inhibited [57]. A previous study showed that Zn^2+^ controlled interferon (IFN)-gamma gene expression in T cells by targeting the calcium-independent PKC activator protein 1 (AP-1) pathway [58]. According to the findings of these studies, Zn^2+^ may influence the immune system by controlling PKC activation in immune cells.

### 3.4. Zn^2+^ Metalloenzymes Regulate Apoptosis

Apoptosis is a highly regulated form of programmed cell death. Normal apoptosis has been proven to be beneficial for maintaining turnover and homeostasis in humans. Caspase, a Zn^2+^-dependent proteolytic enzyme, plays an important role in apoptosis. In response to both external and internal apoptotic signaling factors, the initiator caspases (e.g., caspase 8 and 9) are activated, which in turn mediate the activation of executioner caspases (e.g., caspase 3). Executioner caspases ultimately cleave key autophagy proteins, leading to cell death [59]. 

Zn^2+^ has been reported to control cell apoptosis, which may be through the regulation of caspase activity. Zn^2+^ can directly inhibit enzyme activation by binding to the cysteine in the active site and the second site of caspase 8, resulting in the inhibition of dimer formation. However, in caspase 6, instead of binding to the active site, Zn^2+^ binds to an exosite including lysine, glutamic acid, and histidine residues far from the active site, inhibiting enzyme activation by maintaining an inactive conformation [60,61]. By binding to amino-acid residues at key active sites, increased Zn^2+^ prevents upstream caspase 9 activation, interrupting programmed cell death and triggering the survival and proliferation of damaged cells [60]. In a mouse model of allergic asthma, Zn^2+^ deficiency reduced the inhibition of caspase 3, leading to increased levels of active caspase 3, causing cell apoptosis. Although Zn^2+^ supplementation prevents the programmed cell death induced by various stimuli [62,63], based on the above research, a normal Zn^2+^ status is critical for the regulation of cell apoptosis via the appropriate inhibition of caspase activity. 

### 3.5. Zn^2+^ Metalloenzymes Regulate Other Physiological Processes

Carbonic anhydrase (CA) is a Zn^2+^ metalloenzyme that catalyzes the conversion of carbon dioxide and bicarbonate in the presence of Zn^2+^. When Zn^2+^ ion binds to the luminal binding site of the active site, carbonic anhydrase can play a catalytic role. In α-, γ-, and δ-CAs, Zn^2+^ binds to three histidine residues and a hydroxide ion in the active site. In type I β-CAs, there are two cysteine residues; one histidine residue and a hydroxide ion bind with Zn^2+^, while an aspartate residue replaces the hydroxide ion as the fourth Zn^2+^ ligand in type II β-CAs [64]. Zn^2+^ has been used as an inhibitory target for a wide range of carbonic-anhydrase inhibitors [65]. CA participates in physiological processes related to the regulation of pH and carbon-dioxide balance, such as facilitating the decomposition of carbonic acid in the lungs into carbon dioxide and water to aid respiration, coordinating the reabsorption function of the kidney, and maintaining the synthesis and secretion of hydrochloric acid in the stomach. Since CA is widely present in almost all tissues of mammals, the Zn^2+^ regulation of enzyme activity is crucial. A significantly reduced CA activity in tongue epithelium was identified in rats with a Zn^2+^-deficient diet, which may be responsible for loss of taste [66]. A clinical study revealed that a low-Zn^2+^ diet significantly reduced erythrocyte CA activity, which in turn impaired heart and respiratory function during exercise [67].

The alkaline phosphatases (APs) are a group of isoenzymes that catalyze the hydrolysis of phosphate monoesters and promote the synthesis of DNA [68]. The AP in the intestine, placenta, and germinal tissue is tissue specific, while the AP in the circulatory system is not. There are two Zn^2+^-binding sites and one magnesium-binding site closely spaced at the AP active center. The AP activity reaches its maximum when Zn^2+^ occupies all three metal-binding sites [69]. Zn^2+^ concentrations in the normal range may provide protection for AP to function physiologically. Sadighi et al. demonstrated that Zn^2+^ supplementation caused an increase in AP activity in bone [70]. While there was a significant reduction in the activity and level of AP in the sera of rats fed with a low Zn^2+^ diet, the growth rate was slower than that of rats fed with a normal Zn^2+^ diet [71,72]. In addition, Japan’s Practical Guidelines propose that, apart from liver disease, osteoporosis, chronic kidney disease, and diabetes, a reduction in serum AP has been found to be one of the criteria for Zn^2+^ deficiency [73].

In conclusion, Zn^2+^ can cause an enzyme to gain or lose catalytic activity by critically influencing its structure in a manner that modulates its catalysis or by preventing the binding of the substrate to the enzyme’s active site. The maintenance of Zn^2+^ concentrations within a normal range is beneficial for enabling these Zn^2+^ metalloenzymes to play appropriate catalytic roles in physiological processes.

## 4. Zn^2+^ Metalloenzymes and Diseases

Since Zn^2+^ plays a critical role in regulating the structure and activity of Zn^2+^ metalloenzymes, Zn^2+^ deficiency can lead to various diseases by affecting the function of these enzymes. Due to the fact that previous studies have found that Zn^2+^ deficiency has an impact on human growth and gonadal development, an increasing number of studies have begun to focus on the relationship between Zn^2+^ deficiency and human disease development and how to use Zn^2+^ supplementation to treat these diseases. We have summarized the effects of Zn^2+^ on different diseases through the regulatory effects of Zn^2+^ metalloenzymes (Table 1).

### 4.1. SODs in Cardiovascular Diseases, ALS, and AD

**Figure 2:**
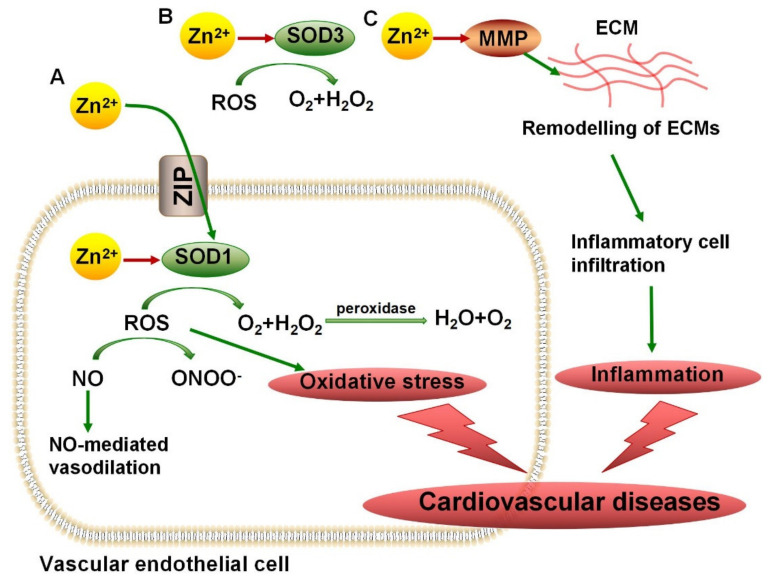
The protective and stimulative mechanism of Zn^2+^ on cardiovascular diseases. (**A**) Zn^2+^ enters cells via ZIP, binds with SOD1 to promote ROS breakdown and protects blood vessels from oxidative stress. ROS promotes the conversion of NO to ONOO^−^. NO is an important component responsible for vasodilation. SOD1 enhances the decomposition of ROS, and the reduced ROS leads to the reduction of NO conversion which facilitates NO-mediated vasodilation. (**B**) In the extracellular space, Zn^2+^ can increase the activity of SOD3, thereby promoting the conversion of ROS and alleviating the damage of oxidative stress to blood vessels. (**C**) The combination of Zn^2+^ with MMP can enhance the activity of enzymes, promoting the degradation of ECMs and the movement of inflammatory cells to the damaged site, ultimately leading to the damage of vessels due to inflammatory response. ZIP, Zrt- and Irt-like protein; SOD, superoxide dismutase; MMP, matrix metalloproteinase; ECM, extracellular matrix; ROS, reactive oxygen species.

Cardiovascular disease (CVD) is the general term for diseases related to the heart and circulatory system, which have become the main cause of death in the world. An increasing number of studies have identified that chronic inflammation and oxidative stress are risk factors for CVDs. Excessive ROS can damage the structure and function of vascular endothelial cells, affecting the generation and progression of CVDs. Since antioxidant copper/zinc superoxide dismutase (Cu/Zn-SOD) has been well known to balance the ROS inside and outside the cell, various studies have focused on the importance of the SOD-mediated antioxidant system in the antioxidant treatment of CVDs [86]. It has been reported that in SOD1-knockout mice, the level of ROS in blood vessels increased and NO-mediated vasodilation decreased, which made blood vessels more vulnerable to damage [74]. Under the condition of exogenous SOD treatment, hypertension and endothelial relaxation damage can both be alleviated [87]. Zn^2+^ has been reported to be responsible for maintaining SOD activity. Zn^2+^-binding sites have been observed in both SOD1 and SOD3. According to an experiment in rats treated with the Zn^2+^ chelator TPEN, TPEN-induced Zn^2+^ depletion led to an increase in the size of myocardial infarction by inhibiting SOD activity [88]. Majewski et al. revealed that resveratrol has been shown to improve blood vessel conditions by increasing SOD activities, which may be induced by an increased concentration of Zn^2+^ [89]. The potential protective mechanism of Zn^2+^ in cardiovascular diseases through controlling SOD activity is shown in Figure 2A,B.

A close relationship between SOD and amyotrophic lateral sclerosis (ALS) has been widely reported. ALS is a type of late-onset degenerative motor neuron disease. Due to its severe lethality, most patients die after about three years because of respiratory failure caused by muscle weakness [90]. It has been reported that about 25% of familial ALS cases are related to a SOD mutation that causes SOD to have a low affinity for Zn^2+^, reducing antioxidant function, and the accumulation of superoxide damages motor neurons and stimulates a neuronal apoptosis cascade [75]. A previous study has demonstrated that SOD mice with a mutation reducing Zn^2+^ affinity survived longer under moderate Zn^2+^ supplementation than those with a Zn^2+^-deficient diet, while excessive Zn^2+^ supplementation can inhibit copper absorption, leading to an increased incidence of anemia and early death [91]. The in vitro experiments revealed that wild-type SOD could also induce motor-neuron apoptosis under the condition of Zn^2+^ deficiency [92]. Since SOD activity plays a key role in the occurrence and progression of ALS, regulating SOD activity using Zn^2+^ may become a therapeutic target. Recently, McAllum et al. found that treatment with a Zn^2+^ metal complex increased the motor function and survival rate of SOD1-mutant mice with low Zn^2+^ affinity, which provided support for the further exploration of the therapeutic effect of Zn^2+^ supplementation on ALS [93].

Oxidative stress has been shown to be a risk factor for neurodegenerative diseases. In addition to ALS, SOD is also involved in the pathogenesis of Alzheimer’s disease (AD). AD is an age-dependent form of dementia with cognitive decline. The formation of soluble amyloid beta (Aβ) oligomer is critical for the pathogenesis of AD, which impairs synaptic and cognitive function. It has been reported that, in animal models, a reduction in SOD can induce Aβ deposition by enhancing oxidative stress, thus leading to cognitive disorders. In patients with AD, there is a significant decrease in the level and activity of SOD compared to that in healthy people [76,77]. As an increase in the central Zn^2+^ concentration promotes the deposition of Aβ, while a low serum Zn^2+^ level may promote the development of AD by triggering depression, the effect of Zn^2+^ disturbance on AD is complex. However, Greenough et al. revealed that presenilin proteins could maintain the activity of SOD1 by promoting Zn^2+^ uptake, inhibiting the accumulation of Aβ [94].

### 4.2. MMPs in Vascular Diseases

Vascular disease is a range of diseases involving vascular dysfunction, including hypertension, atherosclerosis, vascular inflammation, etc. Endothelial dysfunction, aberrant angiogenesis, and abnormal cell survival can lead to vascular diseases [95]. Matrix metalloproteinases (MMPs) have been reported to be responsible for angiogenesis and the reconstruction of vascular tissues. Alterations in MMP activity can lead to uncontrolled inflammatory responses, tissue remodeling, and cell migration [78]. Previous studies have demonstrated that the upregulation of MMP1 and MMP9 activity was significantly positively correlated with the degree of inflammation and organ damage [96,97]. Increased MMP activity can promote the degradation of elastin in the extracellular matrix, resulting in decreased vascular elasticity and thus promoting the progression of hypertension [98]. Since MMPs are Zn^2+^ metalloproteinases, the degree of Zn^2+^-ion binding to Zn^2+^-binding sites determines the activity of MMPs. Since highly active MMPs can remodel ECM and promote the movement of inflammatory cells, Zn^2+^ may aggravate the inflammatory responses of vascular tissues by increasing the activity of MMPs (Figure 2C). The administration of ellagic acid, a Zn^2+^ chelator, can inhibit the activity of MMP2, reducing its angiogenic role in the formation and migration of vascular endothelial cells, which is conducive to inhibiting abnormal angiogenesis [99]. Due to the angiogenesis induced by MMP activation, which can promote tumor metastasis, the development and selection of matrix metalloproteinase inhibitors (MMPIs) have attracted increasing attention [100]. Blocking the binding site for Zn^2+^ or promoting the binding of drugs to free Zn^2+^ ions may provide two approaches to antagonizing MMP activity.

### 4.3. Phosphodiesterase in Chronic Obstructive Pulmonary Disease

**Figure 3:**
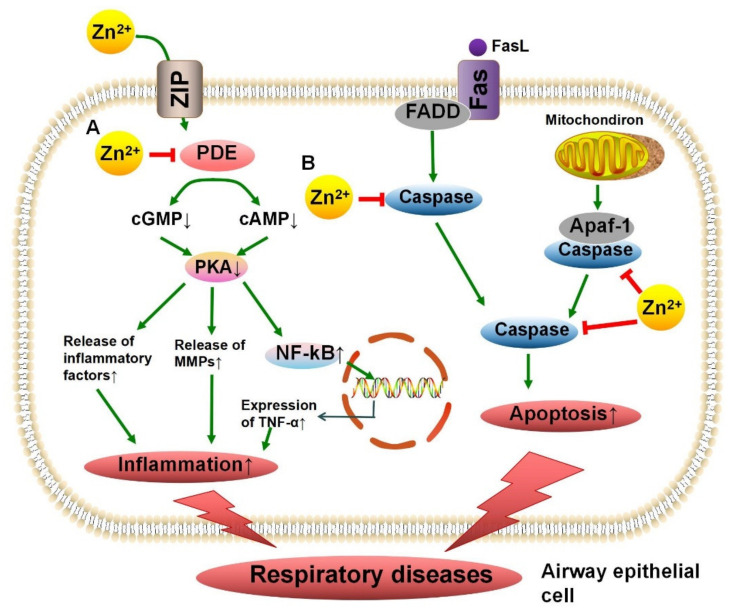
The protective mechanism of Zn^2+^ on respiratory diseases. Zn^2+^ can enter airway epithelial cells via ZIP. (**A**) PDE is responsible for catalyzing the degradation of cGMP and cAMP. Reduced cGMP and cAMP lead to reduced PKA activation, which in turn promotes downstream inflammatory cascades and increased TNF-α expression. Combination of Zn^2+^ and PDE can inhibit the enzyme activity, which reduces the cell damage caused by inflammation. (**B**) Exogenous apoptosis is mediated by FasL and Fas, while endogenous apoptosis is mediated by mitochondria. Both pathways activate initiator and executioner caspases to promote apoptosis. Zn^2+^ protects the airway epithelium by inhibiting caspase activity to prevent excessive cell death. ZIP, Zrt- and Irt-like protein; PDE, phosphodiesterase; FasL, Fas ligand; Fas, tumor necrosis factor receptor superfamily member 6; FADD, Fas-associated death domain; PKA, protein kinase A; NF-Kb, nuclear transcription factor-kappa B.

Chronic obstructive pulmonary disease (COPD) is a progressive irreversible disease that involves airway obstruction with severe airway inflammation. The inhibition of the inflammation of airway cells is the key for treatment [101]. Phosphodiesterase (PDE), a Zn^2+^ metalloenzyme, participates in proinflammatory responses by degrading cAMP, resulting in an increase in TNF-α expression. The inhibition of the activity of PDE4 has been reported to increase the level of cAMP, which in turn inhibits inflammation, relaxes airway smooth muscle, and improves patient symptoms [79]. It has been revealed that decreased serum Zn^2+^ is significantly associated with COPD, and low Zn^2+^ levels may lead to the activation of an inflammatory cascade by inhibiting PDE activity [102]. A recent experiment has shown that Zn^2+^ supplementation can reduce lung macrophages by more than half in mice exposed to cigarette smoke, significantly reducing airway inflammation, which may provide support for micronutrient supplementation for the anti-inflammatory treatment of COPD patients in the future [103]. The mechanism of action of Zn^2+^ in inhibiting the inflammation in the airways by inhibiting PDE activity is shown in Figure 3A.

### 4.4. Protein Kinase C in Immune Diseases

Immune system diseases are diseases caused by an imbalance in the regulation of the immune system or immune response. Recent studies have shown that protein kinase C (PKC) plays an important role in the occurrence and development of human immune diseases. It has been reported that the knockdown of PKCα and PKCθ is significantly associated with decreased T-cell activity. Genome-wide association studies (GWAS) that analyzed people revealed that there was a significant correlation between mutations at the PKC locus and multiple sclerosis, rheumatoid arthritis, and celiac disease [80]. Zn^2+^ is important for PKC’s structure and the regulation of its activity; it can enhance enzyme activity by binding to sites in regulatory regions. In the T cells of mice with immunosuppression caused by hypothyroidism, Zn^2+^ supplementation was beneficial in fighting against a reduction in PKC activity, which is involved in T-cell activation [81]. However, when Zn^2+^ supplementation increased serum Zn^2+^ levels far above normal, Zn^2+^ induced an immunosuppressive effect and inhibited T-lymphocyte activation [104]. In addition, for autoimmune diseases and lymphoma in which increased PKC activity contributes to poor prognosis, the inhibition of Zn^2+^-binding sites may become a target for PKC inhibitors [105,106].

### 4.5. Caspase in Asthma

Asthma is a common chronic respiratory disease characterized by chronic eosinophil infiltration into the airway, with various degrees of airflow restriction accompanied by the apoptosis and exfoliation of airway epithelial cells. A previous study has shown that, compared to that in healthy people, the apoptosis of airway epithelial cells in patients with asthma is significantly increased [107]. Caspase is a critical component of the apoptosis process, and changes in its enzymatic activity affect cell survival. Zn^2+^-binding sites have been discovered in caspase, and Zn^2+^ binding inhibits its activity. A previous study demonstrated that Zn^2+^ acted as a cell-protective agent and inhibited the apoptosis of airway epithelial cells [82]. Roscioli et al. reported that Zn^2+^ deficiency led to increased apoptosis of airway epithelial cells by inhibiting caspase activity [108]. In a mouse model of allergic asthma, a deficiency of Zn^2+^ in the airway epithelium led to changes in active caspase 3 levels and the promotion of apoptosis [62]. It has been revealed that dietary Zn^2+^ supplementation can significantly improve the symptoms of asthma patients, providing a new approach to asthma management [109], in which Zn^2+^ may play an anti-apoptotic role by inhibiting caspase activity (Figure 3B). 

### 4.6. Carbonic Anhydrase in Hypogeusia

The normal activity of CA has been thought to be an important factor in maintaining taste. It has been reported that high levels of CA activity were identified in tongue papillae associated with taste buds [83]. Due to the presence of Zn^2+^-binding sites in CA and the regulatory effect of Zn^2+^ on enzyme activity, various studies have focused on the effects of Zn^2+^ on taste and the treatment of taste disorders [110]. Komai et al. demonstrated that decreased taste sensitivity was associated with decreased CA activity in Zn^2+^-deficient rats [111]. A clinical trial report revealed that exogenous Zn^2+^ supplementation had a certain improvement effect on patients with carbonic anhydrase VI deficiency and hypogeusia [112].

### 4.7. Alkaline Phosphatase in Bone Disorder

In bone, AP can hydrolyze pyrophosphate and increase inorganic phosphate, promoting bone mineralization. Studies have shown that high AP activity is reflected in increased activity of osteoblasts, while low AP activity leads to severe impaired bone mineralization, hypercalcemia, and premature tooth loss [84,85]. The binding of Zn^2+^ to specific binding sites of enzymes is the key to regulating AP activity. It has been reported that incubation with high concentrations of Zn^2+^ can promote the proliferation of osteoblasts, promoting bone formation, which may be caused by an increase in AP activity [113]. A clinical study showed that Zn^2+^ supplementation caused an increase in AP activity and a significant improvement in bone healing [114]. In healthy adult men, Zn^2+^ supplementation also stimulated bone formation, with a significant increase in AP activity [115].

## 5. Zn^2+^ and Zn^2+^ Metalloenzymes in Cancer

An increasing number of studies have confirmed that Zn^2+^ deficiency promotes the occurrence and development of tumors [116]. We have reported that Zn^2+^ deficiency may lead to the dysregulation of important physiological processes in the body, DNA damage, and microRNA expression, resulting in the promotion of cancer [117]. Since the majority of Zn^2+^ metalloenzymes are involved in regulating these physiological processes, Zn^2+^ deficiency may affect tumor progression by controlling the activity of enzymes. Previous research has revealed that a decrease in SOD activity caused by Zn^2+^ deficiency led to oxidative stress, which could induce and maintain the state of cancer cells by impairing DNA stability [118]. Zn^2+^ supplementation may improve the antitumor therapy of non-small-cell lung cancer by increasing ROS activity [119]. In addition, Zn^2+^ deficiency can reduce the function of innate and adaptive immune cells, therefore helping tumor cells to evade immune surveillance [116]. In a previous study, we found that Zn^2+^-deficiency-induced inflammation facilitated the development of esophageal cancer [120]. Taccioli et al. demonstrated that Zn^2+^ supplementation could ameliorate the inflammation in cancer [121]. Although the promoting effect of Zn^2+^ deficiency on tumor development has been comprehensively explored, there is a lack of research on the regulatory effect and influence of Zn^2+^ deficiency on enzymes during tumorigenesis. As Zn^2+^ supplementation has been gradually applied in nutritional support therapy for cancer patients, further exploration of the regulation of enzyme activity by Zn^2+^ deficiency in cancer may provide comprehensive guidance for the management of Zn^2+^ concentrations.

## 6. Conclusions

In this review, we summarized the pathways of Zn^2+^ absorption and metabolism, and the roles and potential mechanisms of Zn^2+^ and Zn^2+^ metalloenzymes in normal physiology and diseases. Zn^2+^ can act as a structural component or catalyst to regulate the activity of Zn^2+^ metalloenzymes, which are involved in antioxidant, anti-inflammatory, immune-response, and apoptotic processes. A deficient or excessive Zn^2+^ intake leads to changes in enzyme activity and affects the occurrence and development of related diseases, which may provide a novel insight for the regulation of enzyme activity and nutritional treatment of diseases. We hope that the comprehensive understanding and in-depth discussion of the mechanisms by which Zn^2+^ regulates metalloenzymes, the effects of Zn^2+^ metalloenzymes on human diseases, and their associated mechanisms will provide new micronutrient supply strategies for the prevention and treatment of human diseases.

## Figures and Tables

**Table 1:** The list of Zn^2+^ metalloenzymes and related human diseases.

Zn^2+^ Metalloenzyme	Zn^2+^ Binding Sites	The Effect of Zn^2+^ on Zn^2+^ Metalloenzyme	Related Diseases	Ref
Copper/Zinc superoxide dismutase	Three histidine residues and one aspartic acid	Activator	CVDs, ALS and AD	[36,74,75,76,77]
Matrix metalloproteinase	Three histidine residues of the catalytic domain	Activator	Vascular diseases	[45,46,78]
Phosphodiesterase	Two histidine and two aspartic acid residues	Inhibitor	COPD	[52,79]
Protein kinase C	Cysteine residues in the regulatory domain	Activator	Immune diseases	[55,80,81]
Caspase	Caspase-6: one lysine, one glutamic acid and one histidine residue out of the active site; Caspase-8: one cysteine in the active site and the second binding site is unknown	Inhibitor	Asthma	[60,61,82]
Carbonic anhydrase	α-, γ-, and δ-CAs: three histidine residues and a hydroxide ion Type I β-CAs: two cysteine residues, one histidine residues, and a hydroxide ion Type II β-CAs: two cysteine residues, one histidine, and one aspartate residues	Activator	Hypogeusia	[64,83]
Alkaline phosphatase	Three metal binding sites in active center	Activator	Bone disorder	[69,84,85]

CVD: cardiovascular disease; ALS: amyotrophic lateral sclerosis; AD: Alzheimer’s disease; COPD: chronic obstructive pulmonary disease.
